# Nasal Osteochondromyxoma Without Carney Complex: A Case Report and a Literature Review

**DOI:** 10.7759/cureus.64223

**Published:** 2024-07-10

**Authors:** Mohammed S Alahmari, Abdulaziz M Alshahrani, Salmah M Alharbi, Mohammed S Alzahrani, Ali Asiry, Waleed Alghamdi, Mohammad Al-Ahmari, Ali Alzarei

**Affiliations:** 1 Otolaryngology-Head and Neck Surgery, Aseer Central Hospital, Abha, SAU; 2 Otolaryngology-Head and Neck Surgery, Armed Forces Hospital Southern Region, Khamis Mushait, SAU

**Keywords:** nasal sinus tumor, benign tumor of bone, unilateral nasal mass, carney complex, osteochondromyxoma

## Abstract

Osteochondromyxoma (OMX) is an extremely rare bone tumor and has been classified by the World Health Organization as a benign chondrogenic bone tumor. The tumor can be associated with Carney complex which is a rare autosomal dominant syndrome. The clinical presentation of the patient depends primarily on the location and the size of the tumor. It has an excellent prognosis with complete surgical excision. Here, in this case, we present a young female patient diagnosed with OMX without carney complex and underwent complete endoscopic surgical excision.

## Introduction

Osteochondromyxoma (OMX) is a very rare bone tumor that affects the diaphysis of long bones as well as paranasal sinuses and nasal bones [1]. Few cases have been reported in the literature. It can be part of the Carney complex, sporadic or congenital as suggested by Carney et al. [2]. OMX has been classified by the World Health Organization as a benign chondrogenic bone tumor [3]. Carney complex is a rare autosomal dominant syndrome characterized by skin lesions, abnormal endocrine activity, and benign and malignant tumors. OMX is presented in 1% of Carney complex patients [4]. It is suggested that mutations in the PRKAR1A gene are the cause of the complex, so along with first-degree family history of the complex they are considered as criteria for diagnosis [5].

Given the rarity of the tumor, few cases have been reported worldwide, its association with a rare syndrome and its unusual presentation, we present a case of OMX of the nasal cavity without the Carney complex.

## Case presentation

A 14-year-old girl presented to the clinic with bilateral nasal obstruction on the right side and obstructive sleep-disordered breathing (snoring and apneic spells during sleep). There were no aggravating or relieving factors. She did not report any other nasal, orbital, or ear symptoms. She had a history of nasal trauma in early childhood. The patient denied experiencing pain, sweating, fever, or loss of appetite. Her past medical history was unremarkable, and her family history was negative for similar presentations.

**Figure 1:**
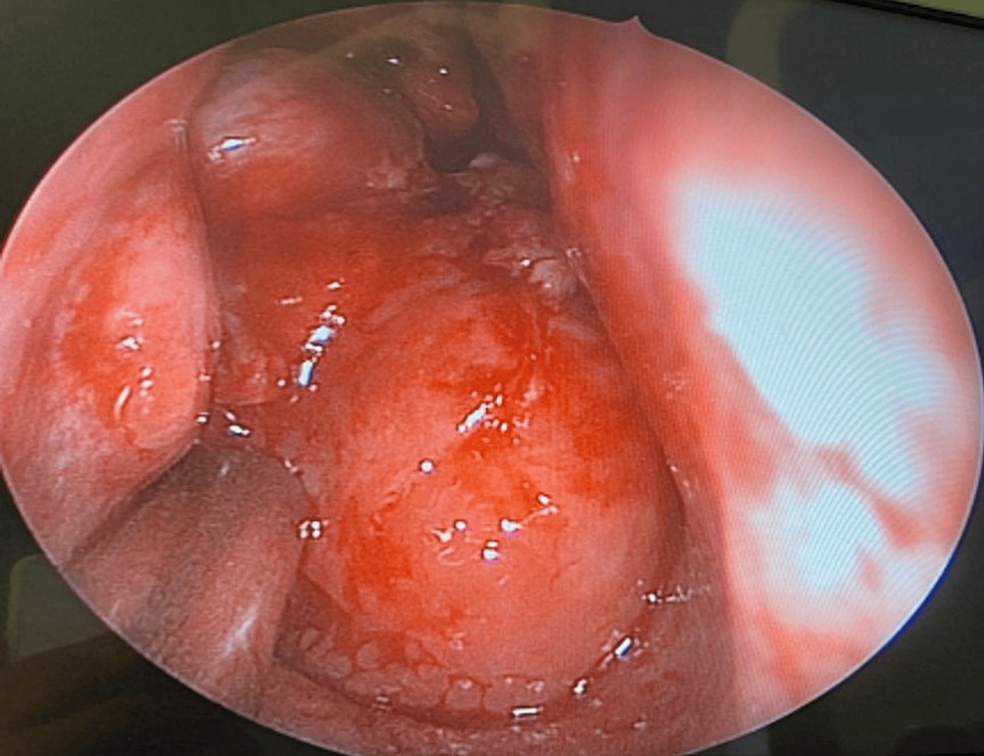
Endoscopic view showing the tumor completely obstructing the right nasal cavity.

On examination, a large smooth mass originating from the lateral nasal wall with normal bleeding tendency was observed fully occupying the right nasal cavity. Other exams were unremarkable (Figure 1).

**Figure 2:**
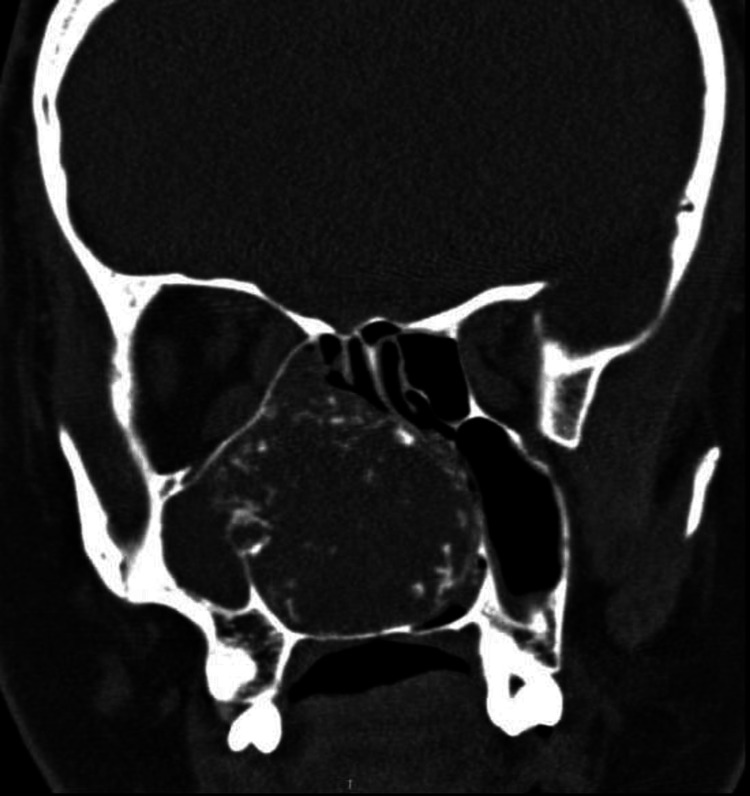
Computed tomography scan with contrast of the paranasal sinus showing the mass fully occupying the right nasal cavity, severe deviation of the nasal septum to the left, and opacification of the right maxillary sinus.

A computed tomography scan with contrast of the paranasal sinus showed a large isodense mass with multiple hyperdense foci fully occupying the right nasal cavity, without invasion of adjacent structures or bony erosion. A deviated nasal septum to the left was noted, along with opacification of the right maxillary sinus (see Figure 2).

**Figure 3:**
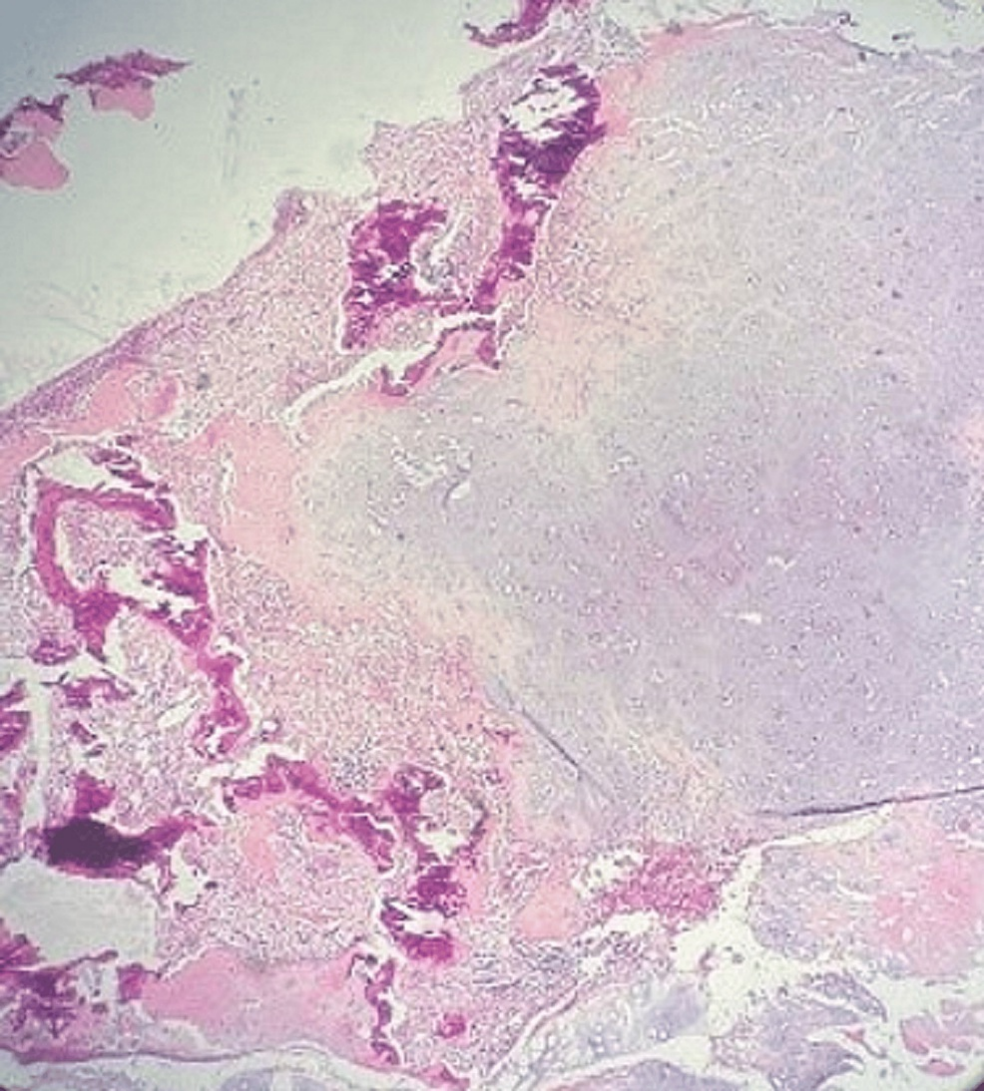
The lesion is composed of multiple fragments of predominantly mature cartilage with foci of atypia in certain areas. Mature lamellated bone, spindle cells, and focal areas of myxoid change are all observed.

A biopsy under local anesthesia was taken from the mass, but the results were inconclusive. Therefore, the patient was consented and taken to the operating room for minimally invasive endoscopic sinus surgery (functional endoscopic sinus surgery, or FESS). The tumor was completely excised without the need for medial maxillectomy. A subsequent biopsy confirmed the diagnosis of OMX (see Figure 3).

The surgery and post-operative course were uneventful, and the patient was discharged home. She was scheduled for follow-up in an outpatient setting by the appropriate specialties.

## Discussion

OMX can affect both males and females, with no accurate male-to-female ratio reported in the literature. It can be discovered at any age but classically affects children before the age of two [6], although cases have been reported at birth [7,8]. Characteristically, OMX affects the diaphysis of long bones, nasal bone as well as paranasal sinuses but it can affect other bones such as temporal bone and chest wall [9,10]. OMX can be sporadic, congenital, or a part of the Carney complex or syndrome. Major criteria for diagnosing this complex include myxoma, skin lesions, abnormal endocrine activity, thyroid cancer, and OMX. The patient must have at least two of the criteria to be diagnosed with the complex. The patient presented in this case did not meet the criteria for the diagnosis. It is suggested that mutations in the PRKAR1A gene are the cause of the complex, so along with first-degree family history of the complex they are considered as criteria for diagnosis [5]. OMX is presented in 1-2% of Carney complex patients [11]. OMX is a benign painless tumor but locally aggressive. The tumor may not be discovered if not surrounded by vital structures or not large enough to cause edema and mass effect. So, the presentation of OMX depends mainly on the site and the size of the tumor [12]. Keeping a high index of suspicion in addition to the appropriate imaging and biopsy when evaluating a unilateral nasal mass is very important. Complete surgical excision of the tumor is the current standard of care with the possibility of recurrence with incomplete excision. The prognosis with complete resection is favorable as there is no report of malignant transformation or metastasis [13].

## Conclusions

Although OMX is a very rare tumor, it should be considered as a differential diagnosis of nasal and paranasal sinuses tumor. Complete surgical resection is the gold standard management with an excellent prognosis. Long-term follow-up is needed as the patient may meet the diagnostic criteria in the future. Multidisciplinary team evaluation is strongly recommended.
